# Brain Responses to Hypnotic Verbal Suggestions Predict Pain Modulation

**DOI:** 10.3389/fpain.2021.757384

**Published:** 2021-12-23

**Authors:** Carolane Desmarteaux, Anouk Streff, Jen-I Chen, Bérengère Houzé, Mathieu Piché, Pierre Rainville

**Affiliations:** ^1^University of Montréal, Montréal, QC, Canada; ^2^University Institute of Geriatrics of Montréal, Montréal, QC, Canada; ^3^University of Québec in Trois-Rivières, Trois-Rivières, QC, Canada

**Keywords:** hypnosis, suggestion, pain, Predictive Coding Model, fMRI

## Abstract

**Background:** The effectiveness of hypnosis in reducing pain is well supported by the scientific literature. Hypnosis typically involves verbal suggestions but the mechanisms by which verbal contents are transformed into predictive signals to modulate perceptual processes remain unclear. We hypothesized that brain activity during verbal suggestions would predict the modulation of responses to acute nociceptive stimuli.

**Methods:** Brain activity was measured using BOLD-fMRI in healthy participants while they listened to verbal suggestions of HYPERALGESIA, HYPOALGESIA, or NORMAL sensation (control) following a standardized hypnosis induction. Immediately after the suggestions, series of noxious electrical stimuli were administered to assess pain-related responses. Brain responses measured during the suggestions were then used to predict changes in pain-related responses using delayed regression analyses.

**Results:** Listening to suggestions of HYPERALGESIA and HYPOALGESIA produced BOLD decreases (vs. control) in the parietal operculum (PO) and in the anterior midcingulate cortex (aMCC), and increases in the left parahippocampal gyrus (lPHG). Changes in activity in PO, aMCC and PHG during the suggestions predicted larger pain-evoked responses following the HYPERALGESIA suggestions in the anterior cingulate cortex (ACC) and the anterior insula (aINS), and smaller pain-evoked responses following the HYPOALGESIA suggestions in the ACC, aMCC, posterior insula (pINS) and thalamus. These changes in pain-evoked brain responses are consistent with the changes in pain perception reported by the participants in HYPERALGESIA and HYPOALGESIA, respectively.

**Conclusions:** The fronto-parietal network (supracallosal ACC and PO) has been associated with self-regulation and perceived self-agency. Deactivation of these regions during suggestions is predictive of the modulation of brain responses to noxious stimuli in areas previously associated with pain perception and pain modulation. The response of the hippocampal complex may reflect its role in contextual learning, memory and pain anticipation/expectations induced by verbal suggestions of pain modulation. This study provides a basis to further explore the transformation of verbal suggestions into perceptual modulatory processes fundamental to hypnosis neurophenomenology. These findings are discussed in relation to predictive coding models.

## Introduction

Many psychological interventions influence pain experiences. The effectiveness of hypnosis in reducing pain is well-supported by the scientific literature (1), making it a powerful candidate for pain modulation in a variety of contexts. Research continues to provide insight into the interplay between hypnotic induction and changes in the experiential field and in brain activity (2–6). Different explanatory models have flourished over the last decades in an effort to explain brain correlates of experiential and behavioral changes following hypnotic induction and suggestions. However, the mechanisms by which verbal information modulates perception, perceived self-agency, or behavior remain largely unexplored. Transformation of words into a pain modulatory effect has yet to be explained, and understanding such processes may provide meaningful insights into brain-body interactions and embodied cognition (7–9).

To explain language processing in situation modeling, Cayol and Nazir (10) proposed that the brain acts as an emulator that “captures the relationship between an action and its sensory consequences.” Anchored in feedforward models of motor control (11), this emulator generating predictions that guide the processing of sensory input provides a model of the mechanisms underlying the neural activity behind verbal integration that we may observe in hypnosis. By illustrating how language affects perception processes, this model provides a potential basis for relating semantic processing to learning optimization (12).

Neuroimaging of suggestion effects is a critical step in the study of hypnosis. A primary role of prefrontal and anterior cingulate cortex activity is key in the top-down interpretation of perceptual modulation (13). However, hypnosis studies typically test the effect of hypnotic suggestions on perceptual or motor responses and do not examine specifically the brain processes underlying the encoding of verbal hypnotic suggestions. Other experimental models have been used to study the impact of verbal stimuli on subsequent brain responses. Verbal priming has been described as a contextualization of subsequent perception, modifying not only the behavior, but also an early neural response (14). This provides a potential framework to understand the influence of lexicosemantic processes and contextual/verbal associative learning on pain perception (15).

Various studies demonstrated the effects of verbally-induced psychological states on the experience of pain. Both verbal suggestions with negative valence and pain-related verbal suggestions may increase perceived pain intensity (16). Facilitation of pain and pain-related brain activation further seems to be stronger for words related to pain than for other negative words matched on valence (17). Verbal suggestions may also prime a hypoalgesic effect, leading to a decrease in pain-related brain activity as reported in placebo effects [e.g., (18)]. Inversely, the fear of pain may be induced verbally to produce anticipatory physiological responses through mechanisms at least partly dissociable from classical aversive conditioning processes (19, 20). Verbal suggestions can further minimize (and sometimes reverse) a conditioned nocebo response (21), or enhance conditioning of the nocebo response (22). Corsi et al. (23) also found that the effect of verbal suggestion may outweigh the positive conditioning of motor nocebo effects, supporting a predominant role of verbal suggestion over conditioning in influencing behavior and perception. The enhancement of the placebo effect by verbal suggestion involves brain regions underlying memory-semantic processes (18, 24). Regions such as the lentiform nucleus, parahippocampal complex and superior temporal gyri, are thought to act as a memory-semantic network “likely to sustain the memory of the recent placebo suggestion and the meaning of that suggestion” (18). This contextual association is a plausible explanation of the activation of top-down pain modulation when there is a semantic integration at play (25). Verbal suggestion may thereby prepare and contextualize pain perception in a top-down fashion, through this neural network.

In order to better understand the language processing of hypnotic suggestions, it is essential to separate brain activation underlying verbal encoding from the brain response reflecting the modulatory effects produced by the suggestions. Most studies on hypnosis have focused on downstream modulatory effects rather than the verbal encoding of suggestions. No study has yet directly investigated the brain networks underlying the interaction between these two key moments of the hypnotic experience. The primary goals of this study are to explore brain responses to verbal suggestions for pain modulation delivered following a standardized hypnotic procedure and to test the hypothesis that these responses would predict the changes in brain responses to noxious stimulation. Analyses were designed to reveal brain regions/networks activated by suggestions of pain modulation (hyperalgesia and hypoalgesia vs. neutral suggestions) and those possibly involved differentially in hyperalgesia or hypoalgesia. We hypothesized that responses to suggestions would predict the modulation of brain responses to noxious electrical stimulation. More specifically we expected that brain responses to verbal suggestions of hypoalgesia (vs. neutral suggestions) would predict a decrease in stimulus-evoked activity within pain-responsive regions (e.g., S1, S2, insula and mid/anterior cingulate cortex). In contrast, changes in brain activity during verbal suggestions of hyperalgesia (vs. neutral suggestions) were expected to predict an increase in stimulus-evoked activity within pain-responsive brain regions. This allowed mapping the dynamic process underlying the conversion of verbal suggestion into pain modulatory effects.

## Materials and Methods

### Participants

Recruitment was conducted through the participant registry at the Research Center of the “Institut Universitaire de Gériatrie de Montréal” (CRIUGM) and through advertisement on the campus of Université de Montréal. Exclusion criteria were recent consumption of pain medication (2 weeks prior to the experiment) or medication that could alter pain perception and modulation (e.g., antihypertensive, anxiolytic, antidepressant, and other psychotropic agents), as well as self-reported history of chronic pain, psychiatric and neurological disorders.

From the 33 participants recruited, seven were excluded from the analysis due to incomplete datasets, and two from excessive head motion during scans. The remaining 24 were included in the analyses (13 females and 11 males; mean age: 26.8; SD: ±1.1 y.o.). Participants were asked to stop consuming alcohol during the 24 h preceding the scan, and to refrain from consuming coffee and tea on the day of the experiment. All participants were part of a separate psychophysiological experiment involving similar experimental conditions prior to the present fMRI study (5). All experimental procedures met the guidelines of the latest revision of the Declaration of Helsinki and were approved by the ethics committee of the CRIUGM. All participants provided written informed consent and received monetary compensation for their participation. At the time of the study, the consent form did not include an authorization for the sharing of individual data outside of this research group.

### Experimental Procedure

This study was conducted over two sessions, taking place on different days. On the first session, individual hypnotic suggestibility was evaluated and participants were familiarized with the pain protocol. The second part of the experiment consisted of a brain imaging session in which a structural scan, resting-state arterial spin labeling (ASL) perfusion, and pain-related blood-oxygen-level-dependent (BOLD) fMRI data were acquired. Results of the ASL scans are reported in a separate article (5).

### Pre-scanning Session

Hypnotic suggestibility was assessed with the French version of the Stanford Hypnotic Susceptibility Scale, Form A [SHSS:A; (26)]. The SHSS:A administration is individualized and takes 30–45 min. It constitutes a robust index of suggestibility composed of 12 test items assessing behavioral responses to ideomotor (e.g., hand lowering) and cognitive (e.g., amnesia) suggestions following a hypnotic induction. Participants were not pre-selected based on their hypnotic suggestibility, but the SHSS-A was used to characterize this convenience sample and to verify that the reported pain modulation was consistent with this standardized measure of hypnosis responsiveness (27).

### Brain Imaging Session

#### Scanning Procedure

Imaging data was collected at the Unité de neuroimagerie fonctionnelle (UNF) of the CRIUGM using a 3T Siemens Magnetom TIM Trio magnetic resonance imaging (MRI) system with a 12-channel head coil. Participants were positioned comfortably in the scanner and stabilized with a pelvic strap as well as foam pads to immobilize the head. MRI compatible earphones were used to communicate with the participants and to provide hypnotic suggestion while reducing the noise from the scanner. The entire scanning session lasted ~75 min.

**Figure 1:**
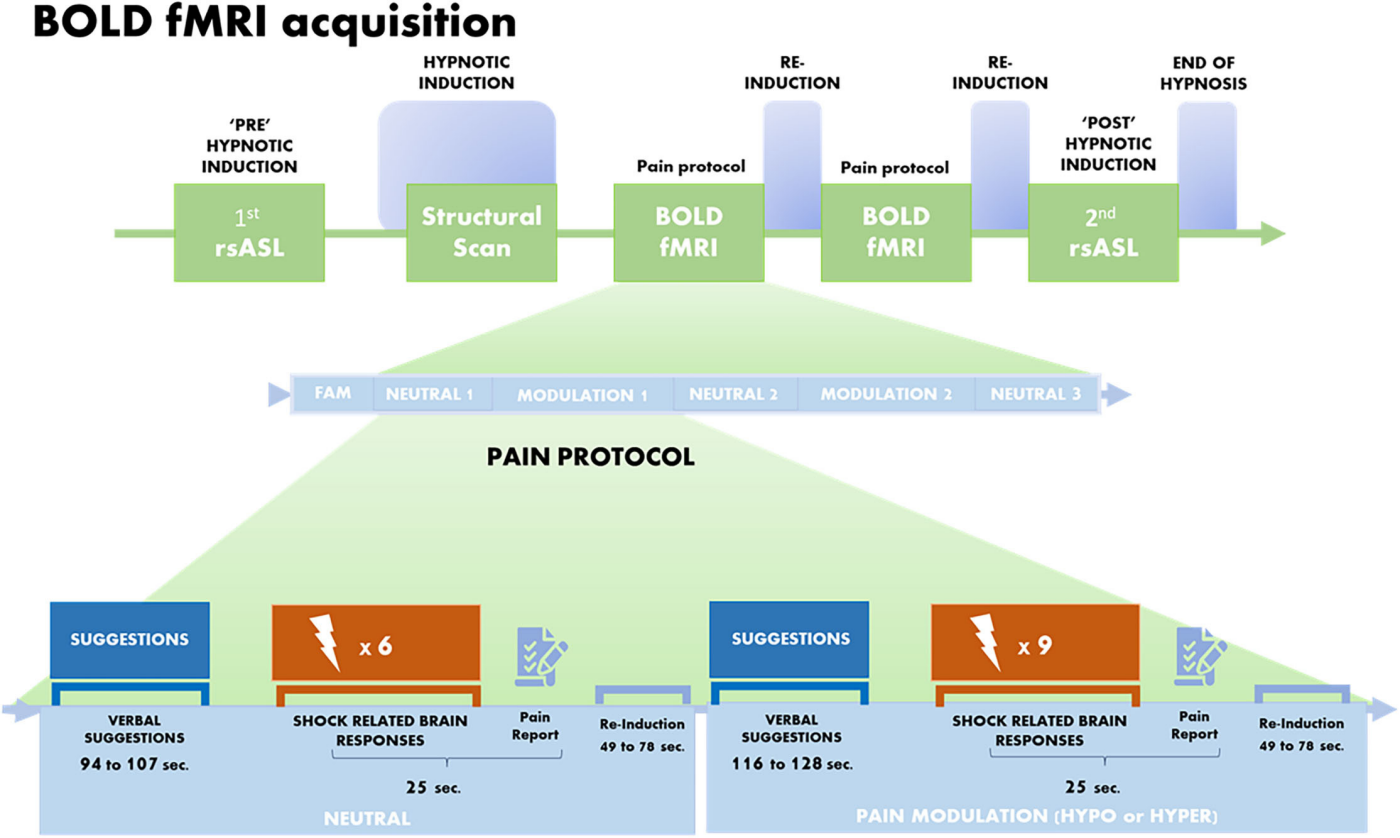
Brain imaging protocol. BOLD fMRI acquisition was performed between two rsASL scans and after the T1 structural scan. Each fMRI run started with a familiarization block (FAM), then included neutral suggestions (Normal sensation) and modulation suggestions of Hypoalgesia or Hyperalgesia (Hypo and Hyper counterbalanced between runs). Each run was composed of an alternation of three neutral and two pain modulation conditions. Each condition involved suggestions to feel pain as one would normally do (Neutral; duration varies between 94 and 107 s) or suggestions to feel more pain (Hyper) or less pain (Hypo) (duration varies between 116 and 128 s; see verbatim in Supplementary Material). Following verbal suggestions, a series of six shocks were delivered in the Neutral condition, and 9 in the pain modulation condition (Hyper or Hypo), for a total of 18 shocks per condition, per run. Each shock lasted 30 ms, with variable ISIs of 6, 9, or 12 s between successive shock. At the end of each series of shocks, participants rated the overall intensity and the unpleasantness of the pain they felt, on a visual analog scale (VAS) converted to values of 0–100.

During the scanning session, a T1-weighted structural MRI scan, two resting-state ASL scans, and two blood oxygen level-dependent (BOLD) scans were acquired (Figure 1). Briefly, the session started with the first resting-state ASL scan acquired in the normal awake state (i.e., pre-hynpotic induction), followed by the initiation of the pre-recorded hypnotic induction taken from the SHSS-A protocol (14 min). The anatomical scan was then launched 8 min into the induction, such that the induction was completed at the end of the anatomical scan. Then, two BOLD fMRI scans were acquired during hypnosis as part of the pain protocol. The imaging session concluded with the second ASL scan acquired during hypnosis (“POST” induction; 6 min) and followed by suggestions to end hypnosis and recover normal alertness (Figure 1).

#### Hypnotic Induction

A pre-recorded hypnotic induction based on the SHSS:A was delivered via earphones before and during the anatomical scan, for ~14 min. Participants were first asked to visually fixate on a cross displayed on the scanner screen visible through a mirror attached to the head coil. A brief psychoeducation about hypnosis ensued. Then, participants were invited to pay attention to the voice and to the suggestion and to comfortably enter into a relaxed state by letting their body become heavy and relaxed. Participants were also encouraged to concentrate on the present moment, as their thoughts might come and go, simply by being attentive to and curious about what is happening. Then, it was suggested that they could pay attention to the voice and to the suggestion, without being distracted by the scanner noise. Relaxation is further enhanced by a count (1–20) suggested to deepen the hypnotic state. Eyelids typically close during the induction procedure but an explicit instruction to close the eyes was included before the pain protocol started. After each fMRI scan, additional suggestions taken from the induction procedure (e.g., count) were given to suggest the maintenance or the deepening of the hypnotic state. Subjective reports of the experience of automaticity and hypnotic depth were taken outside of the pain fMRI runs, as described in our previous report of the rsASL results (5).

#### Painful Stimulation and Suggestions for Pain Modulation

Transcutaneous electrical stimulation was delivered with a Grass S48 square pulse stimulator (Grass Medical Instruments, Quincy, MA, USA), through a constant-current stimulus-isolation unit and a radio-frequency (RF) filter. The stimulation consisted of a 30-ms train of 10 × 1 ms pulse, delivered on degreased skin over the retromalleolar path of the right sural nerve using a pair of surface Ag/AgCl electrodes (diameter 8 mm; Biopac EL258RT, Biopac Systems) with an interelectrode distance of 15 mm. Stimulus intensity was adjusted individually prior to the scans to induce a reliable nociceptive flexion reflex (28) and to produce moderate pain in each participant. This individual calibration procedure was applied to reduce the risk of floor or ceiling effects in response to hypoalgesic and hyperalgesic suggestions. Reflex responses were not readily analysable due to technical problems and MRI-induced noise in the EMG signal. This measure is not discussed further.

The pain protocol started with a brief block of familiarization (4 electrical stimuli), followed by an alternation of three neutral and two pain modulation conditions. The pain modulation condition, consisting of either hyperalgesia (Hyper) or hypoalgesia (Hypo), were administered over two separate runs in a counterbalanced order across participants (Figure 1). Participants received suggestions to feel pain as they would normally do during the control condition (Neutral), followed by six painful shocks. During pain modulation conditions, suggestions to feel more pain (Hyper) or less pain (Hypo) were given, followed by nine painful shocks. The duration of the verbal suggestions varied from 94 to 138 s.

Hyperalgesia and Hypoalgesia suggestions involved attention directed to the foot while imagining a transformation into metal, an effective electro-conductive material (Hyper), or rubber, an effective insulator (Hypo). Although this is not indicated in clinical hypnosis, negatively valenced wording qualifying the sensation was used in both conditions to preserve the similarity of the Hyper and Hypo conditions. The two experimental conditions differed essentially in the direction of the suggested pain modulatory effect (see verbatim in Supplementary Material). A total of 18 shocks per condition were therefore administered in each run. Stimuli were delivered with a pseudo-randomized inter-stimulus interval (ISI) of 6, 9, or 12 s. At the end of the series of shocks, participants were asked to open their eyes to rate the intensity (INT) and the unpleasantness (UNP) of pain on a VAS scale converted to values of 0–100. Participants were asked to close their eyes again after the rating.

#### Structural Images

High-resolution (1-mm isotropic voxels) anatomical images were acquired using T1-weighted multi-echo MPRAGE sequence (ME-MPRAGE) with the following parameters: 176 slices per whole brain volume, repetition time = 2,530 ms, 4 echo times = 1.64, 3.50, 5.36, 7.22, 13, and 15 ms combined to form one root mean squared volume, flip angle = 7°, field of view (FOV) = 256 mm, matrix = 256 × 256, parallel imaging with GRAPPA 2, and a bandwidth of 651 Hz/Px. The anatomical scan lasted 6.3 min.

#### Functional Images

Functional scans were acquired using blood oxygen level-dependent (BOLD) protocol (29) with a T2^*^-weighted gradient echo, echo-planar imaging (EPI) sequence (in-plane resolution = 3 × 3 mm; TR = 3000 ms, TE = 20 ms; flip angle = 90; matrix = 74 × 74; FOV = 220 × 220 mm^2^; bandwidth = 2,414 Hz/Px; GRAPPA 2). Each functional run comprised of 400 volumes of 50 interleaved axial slices of 3-mm thickness with a backward tilt of 30 degrees relative to the AC-PC line, covering the entire brain from the vertex of the cortex to the lower brainstem.

### Analysis

#### Behavioral Data

The effect of suggestions on pain evaluation was assessed with paired *t*-tests comparing each pain modulation suggestion (Hypo and Hyper) to the neutral control acquired within the same run. Pain intensity and unpleasantness ratings were strongly correlated with each other within conditions across subjects (*r*'s = 0.76–0.95) so results are reported based on the intensity ratings. Non-parametric correlation between change in pain (Hyper > Neutral, Hypo > Neutral) and SHSS scores were then performed for each condition to verify that pain modulation was consistent with this standardized measurement hypnotic responsiveness.

#### Imaging Data

##### Preprocessing

Image analysis was performed with SPM8 (Statistical Parametric Mapping, Version 8; Wellcome Department of Imaging Neuroscience, London, UK), executed in Matlab 7 (Mathworks, Sherborn, Massachusetts). Functional images were first pre-processed with slice-time correction, and motion corrected by realigning all images to the first image using six-parameter rigid body transformation and re-slicing with fourth degree B-spline interpolation. The BOLD and structural images were spatially normalized to MNI space using unified segmentation-based method, with the normalization parameters determined during the segmentation of the structural images. Spatial smoothing was subsequently applied to the functional images using a 6-mm isotropic full width half maximum (FWHM) Gaussian kernel in order to increase signal-to-noise ratio. A high-pass temporal filter (cut-off = 428 s) and correction for auto-correlation between successive volumes (AR1) were applied to the time series.

##### General Linear Model

First-level analysis was performed using a canonical hemodynamic response function. Analyses were performed using the general linear model (GLM) to obtain parameter estimates of suggestion-related and stimulus-related activity at each voxel and for each suggestion condition and each stimulus.

Verbal suggestions were modeled using a boxcar function. In order to assess separately the prediction from suggestions of Hypo and Hyper to the brain responses to the following pain, eight conditions were included in the model: suggestion for normal sensation in the hypoalgesia run (“Sugg N_Hypo”); shocks following those Neutral suggestion (“N_Hypo_shocks”); suggestion for hypoalgesia (“Sugg Hypo”); shocks following Hypoalgesic suggestion (“Hypo shocks”); suggestion for normal sensation in the hyperalgesia run (“Sugg N_Hyper); shocks following N_Hyper suggestion (“N_Hyper shocks”); suggestion for hyperalgesia (“Sugg Hyper”); shocks following Hyperalgesic suggestion (“Hyper shocks”).

Painful shocks were modeled as instantaneous events at the trial level (i.e., 18 trials per condition per participant).

Additionally, the 6 motion correction parameters (three translational and 3 rotational), as well as the mean signals across voxels from the white matter and the cerebrospinal fluid, were included in the design matrices as nuisance regressors in order to account for possible effects of head movements and to reduce possible physiological noise.

First-level contrast images were then used in second-level (group) analyses to compare the overall effect of the pain modulation conditions (Hyper & Hypo) vs. neutral. Significance was assessed using the False Discovery Rate (FDR) method with a threshold set to *q* = 0.05.

##### Psychophysiological Interaction Analysis

The main contrast between brain responses to hyperalgesic and hypoalgesic suggestions did not reveal any significant effect, suggesting that the direction of the suggestions did not affect brain activity (see results). However, this basic contrast may miss meaningful differences in connectivity. An exploratory functional connectivity analysis (30) was performed on seed regions of interests (ROIs) defined by responses to verbal suggestions (PO, aMCC and PHG) in order to determine whether the connectivity patterns of the modulation suggestions differ as a function of the direction of the suggestions (Hypoalgesia vs. Hyperalgesia).

Volumes from each Hypo and Hyperalgesia run were first concatenated to create a single time-series of volumes where the between-run conditions can be contrasted (Sugg N_Hypo, Shocks N_Hhypo, Sugg Hypo, Shocks Hypo, Sugg N_Hyper, Shocks N_Hyper, Sugg Hyper, Shocks Hyper). Additionally, values of the movement parameters and mean signal from WM and CSF were also concatenated, and a scan regressor was added to account for inter-scan differences (i.e., runs). First-level GLM analysis was then performed to produce individual images, where contrast of “Hyper vs. Hypo,” and the inverse, were used as the effects of interest in the subsequent PPI analysis.

The PO mask was based on a 10-mm sphere centered on the coordinates of the peak observed in the contrast Hyper & Hypo < Neutral. The aMCC mask was based on the combined 10-mm spheres centered on aMCC peaks from the same contrast. The PHG mask was created based on a 10-mm sphere of 2 left PHG seed regions in the reversed contrast Hyper & Hypo > Neutral.

For each ROI, the raw time course of BOLD signal from each subject was extracted from the concatenated volume, and deconvolved using Bayesian estimation to create a time series representing the neural signal in each of these regions. The interaction term (“PPI regressor”) was then generated as the element-by-element product of the condition time course and the deconvolved time course, and convolved back with the HRF to form a predicted PPI time series at hemodynamic response level. The final interaction terms were then entered as regressors in the subsequent 1st level GLM analysis to produce images of contrast estimates showing areas of connectivity to the seed regions varying as a function of verbal suggestion of Hyper vs. Hypo conditions. These contrasts were entered into the 2nd level random effects group analysis (one-sample *t*-test) at a statistical significance level of FDR of *q* = 0.05. Statistical volumes were further explored at *p*-uncorrected <0.001 to protect against type II error.

##### Delayed Regression Analysis

**Figure 2:**
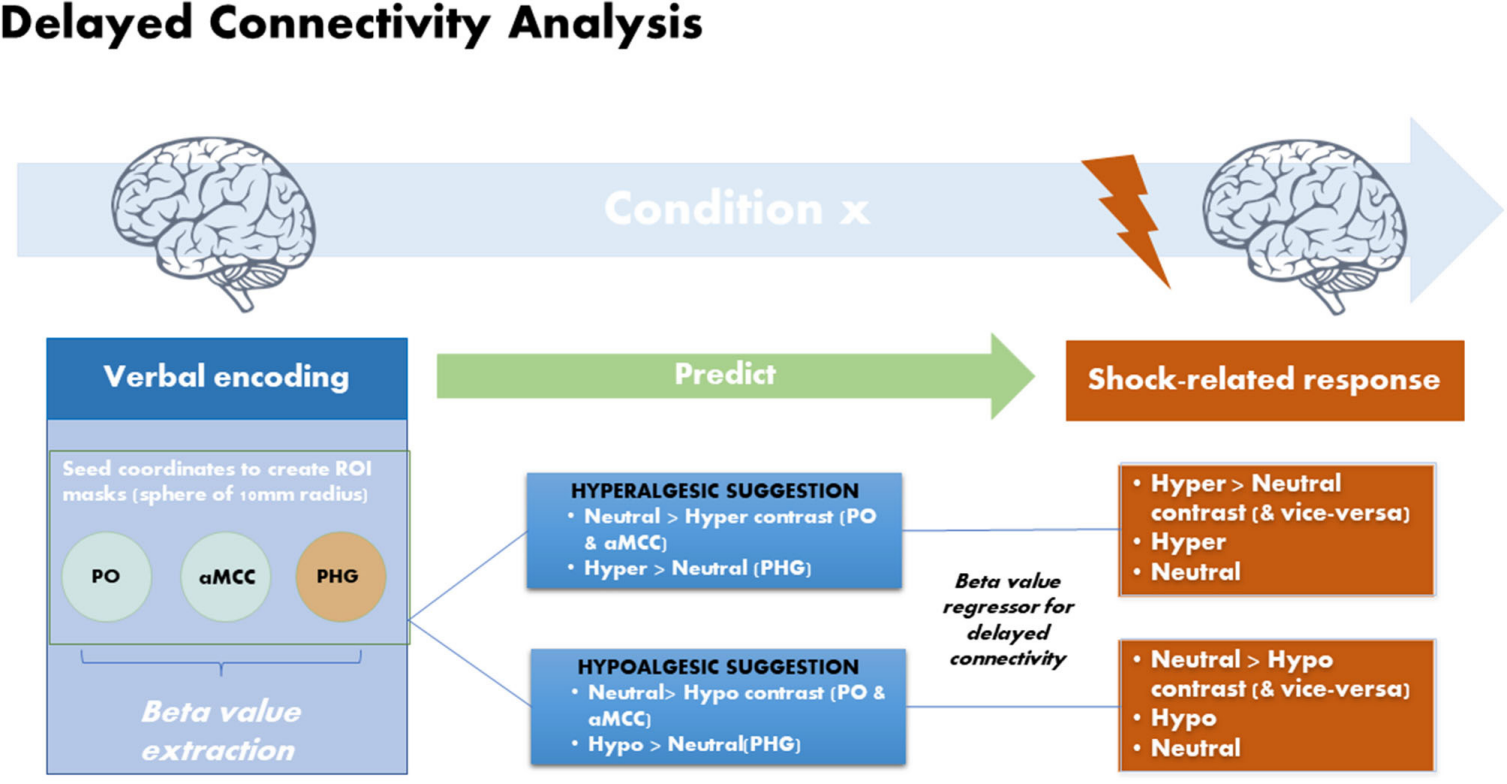
Delayed regression analysis: brain responses measured during the verbal suggestions (seeds) were used to predict changes in shock-related responses using delayed regression analyses. Verbal suggestions of both Hypoalgesia and Hyperalgesia produced BOLD decreases in PO and aMCC and BOLD increases in left PHG. Beta values were extracted from these regions in each subjects separately for the Hyperalgesic and Hypoalgesic suggestions vs. the control condition (Neutral suggestion). The extracted Beta values were then used as subject regressors to predict changes in shock-evoked responses in the corresponding contrasts.

Brain responses to the suggestions were used to predict the modulation of brain responses to the upcoming painful stimulation in a delayed regression analysis using the 3 ROI defined in the PPI analysis (Figure 2). The mean beta weights from the ROIs were extracted using MarsBaR (v.0.44) (31) from the first level contrast in each subject and separately for each modulation condition (Hypo and Hyper) compared to the Neutral control. This effect of the verbal suggestions obtained in each participant and in each modulation condition (Hypo and Hyper) was then used as a subject-regressor to predict the modulation of brain responses to the painful stimulation in the respective contrasts between the Hyper and Hypo conditions vs. the neutral control condition (Figure 2). All effects are assessed at the statistical threshold of FDR *q* = 0.05.

## Results

### Pain Modulation

Pain ratings confirmed that suggestions of hyperalgesia and hypoalgesia produced significant changes in pain perception in the expected direction (Hypo *p* = 0.003; Hyper *p* = 0.01). We computed a *t*-test comparing the magnitude of the absolute change in pain in the two conditions (Hyper mean: 12.3; Absolute Hypo mean: 7.58). This comparison did not reveal significant differences (*t* = 1.8, *p* = 0.76). Thus, we may conclude that the absolute magnitude of pain modulation following hyperalgesic and hypoalgesic suggestions were not significantly different. During Hypo, participants rated pain at an average intensity of 40 (SD: ±21) compared to 46 (SD: ±19) in the control condition (Neutral suggestions). In Hyper, pain was rated at an average of 56 (SD: ±20), compared to 46 (SD: ±19) in the control condition.

SHSS scores varied from 1 to 12 across participants (Mean: 7; SD: ±3.6). Changes in pain intensity (Hyper minus Neutral and Hypo minus Neutral) was proportional to suggestibility scores across individuals (Hyper: *r* = 0.68 *p* < 0.001 and Hypo: *r* = −0.56, *p* = 0.004). These behavioral results confirmed pain modulatory effects consistent with hypnosis.

### Suggestion-Related Brain Activity

**Figure 3:**
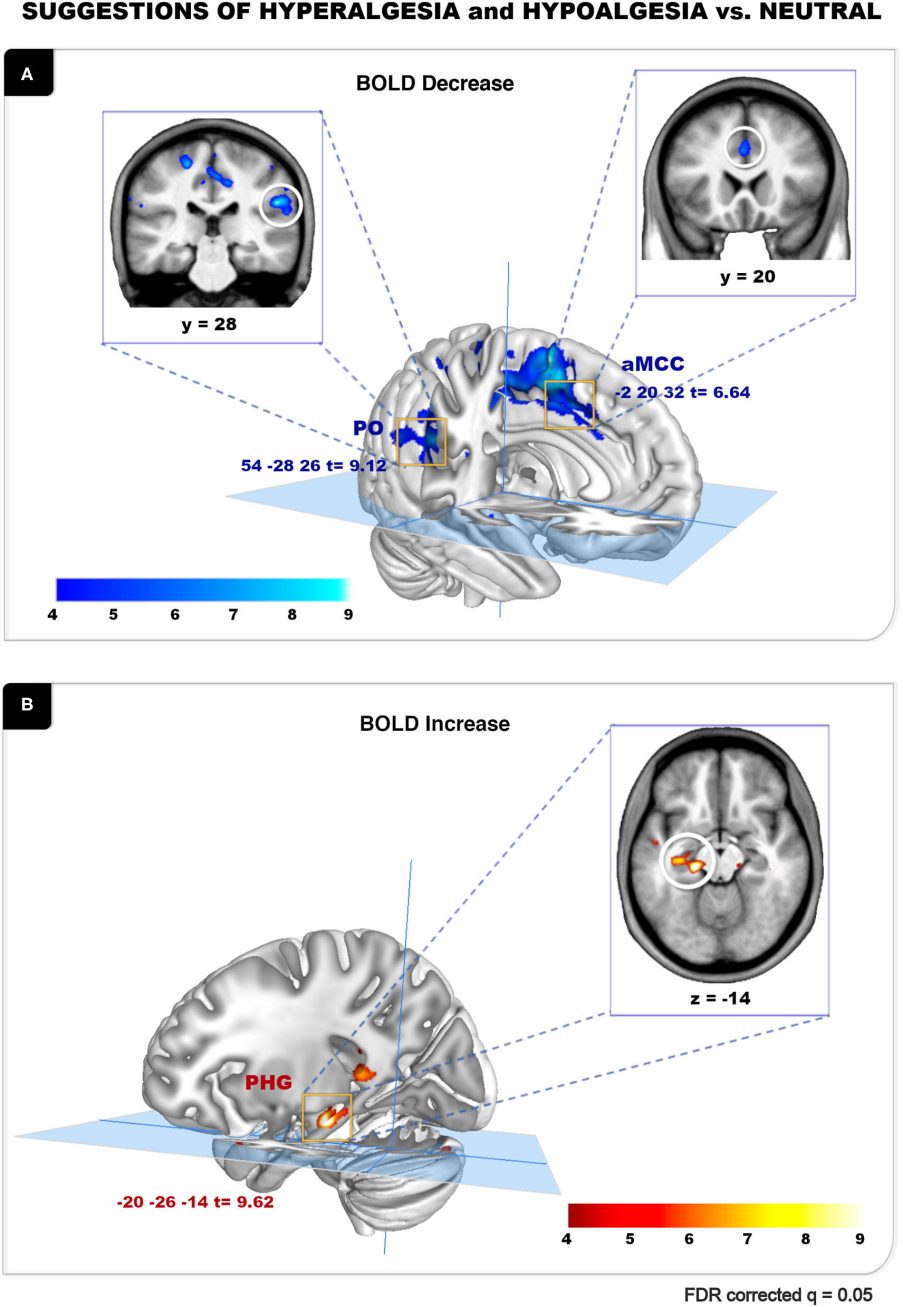
Suggestion of hyperalgesia and hypoalgesia vs. neutral. **(A)** Verbal suggestion-related peak for [(Hyper + Hypo) < Neutral] conditions. Significant BOLD decrease was observed in the right PO (*t* = 9.12) and the aMCC (*t* = 6.64) during suggestions. **(B)** Verbal suggestion-related peaks for [(Hyper + Hypo) > Neutral] conditions. Significant BOLD increase was observed in the lPHG (*t* = 9.62) during suggestions. Results are reported at FDR *q* = 0.05 (also see Table 1).

The analysis of BOLD responses to the verbal suggestions revealed robust effects of the hypoalgesia and hyperalgesia conditions (vs. Neutral control). A general suggestion effect was observed, where the combined Hyperalgesia and Hypoalgesia suggestions were associated with a significant BOLD decrease in the right parietal operculum (PO; peak *t* = 9.12) and in the anterior cingulate cortex (aMCC; peak *t* = 6.64) (Figure 3A; Table 1). Significant BOLD increase was observed during pain modulation suggestions conditions, in the parahippocampal gyrus with the highest peak on the left side (lPHG; peak *t* = 9.62) (Figure 3B; Table 1). Additional peaks are reported in Table 1. Similar effects were observed on the Hyper and Hypo conditions analyzed separately (not reported). A direct contrast between Hyperalgesia and Hypoalgesia suggestions did not yield any significant difference, suggesting a non-specific effect independent of the direction of the suggested modulation.

**Table 1:** Pain modulation suggestion-related increase and decrease in brain activity.

**Brain area**	**Coordinates**			**Local peak *t*-value**
	***x***	***y***	***z***	
**Decrease (Hyper & Hypo** **<** **Neutral)**				
PO	54	−28	26	9.12^*^
aMCC	−2	20	32	6.64
	4	10	40	5.44^*^
	2	22	30	5.25^*^
	−8	14	36	5.01^*^
Supplementary motor area (SMA)	2	−2	48	9.32
	2	−8	48	9.32
	0	−6	64	9.11
	42	−8	42	6.85
	−40	−16	42	6.35
Primary motor cortex (M1)	−22	−24	60	8.95
	24	−22	58	7.92
	−48	−16	56	5.78
Mid cingulate cortex (MCC)	−8	4	42	7.30
	8	6	44	6.06
	14	−22	38	6.02
pINS	32	−24	14	6.50
	−36	−22	20	5.07
aINS	−42	0	10	5.81
	34	8	10	5.11
	−36	4	10	4.93
PCC	14	−30	42	7.24
	−14	−36	42	6.11
S1	28	−32	66	6.48
V1	12	92	−2	5.02
V2	−14	−100	14	6.52
	16	−98	12	5.24
**Increase (Hyper & Hypo** **>** **Neutral)**				
PHG	−20	−26	−14	9.62^*^
	−34	−22	−12	8.20
	−16	−30	−10	6.52^*^
	20	−34	−18	6.47
	30	−34	2	6.43

*All effects are reported at FDR q = 0.05*.

* *ROI seed regions used in the subsequent PPI and delayed regression analysis*.

### Functional Connectivity During Suggestions

PPI analysis was conducted to detect possible differential patterns of connectivity related to the direction of the suggestions. The analyses did not yield significant results for any of the 3 ROIs at the conservative statistical threshold (FDR *q* = 0.05). Connectivity maps were further examined at a more permissive statistical threshold (*p* < 0.001 uncorrected) as an exploratory measure to protect against a possible type II error. In these analyses, the increase in left PHG was found to be more strongly associated with the left nucleus accumbens (NAc) when contrasting Hypoalgesia vs. Hyperalgesia suggestions, and with the left amygdala in Hyperalgesia vs. Hypoalgesia suggestions (additional peaks are reported in Supplementary Table 1).

### Delayed Regression Analysis: Prediction of Shock-Evoked Responses

Results on the delayed regression analysis indicate that changes in activation in the PO, aMCC and PHG during suggestions for pain modulation predicted the magnitude of changes in shock-evoked BOLD response in Hyper and Hypo conditions.

**Figure 4:**
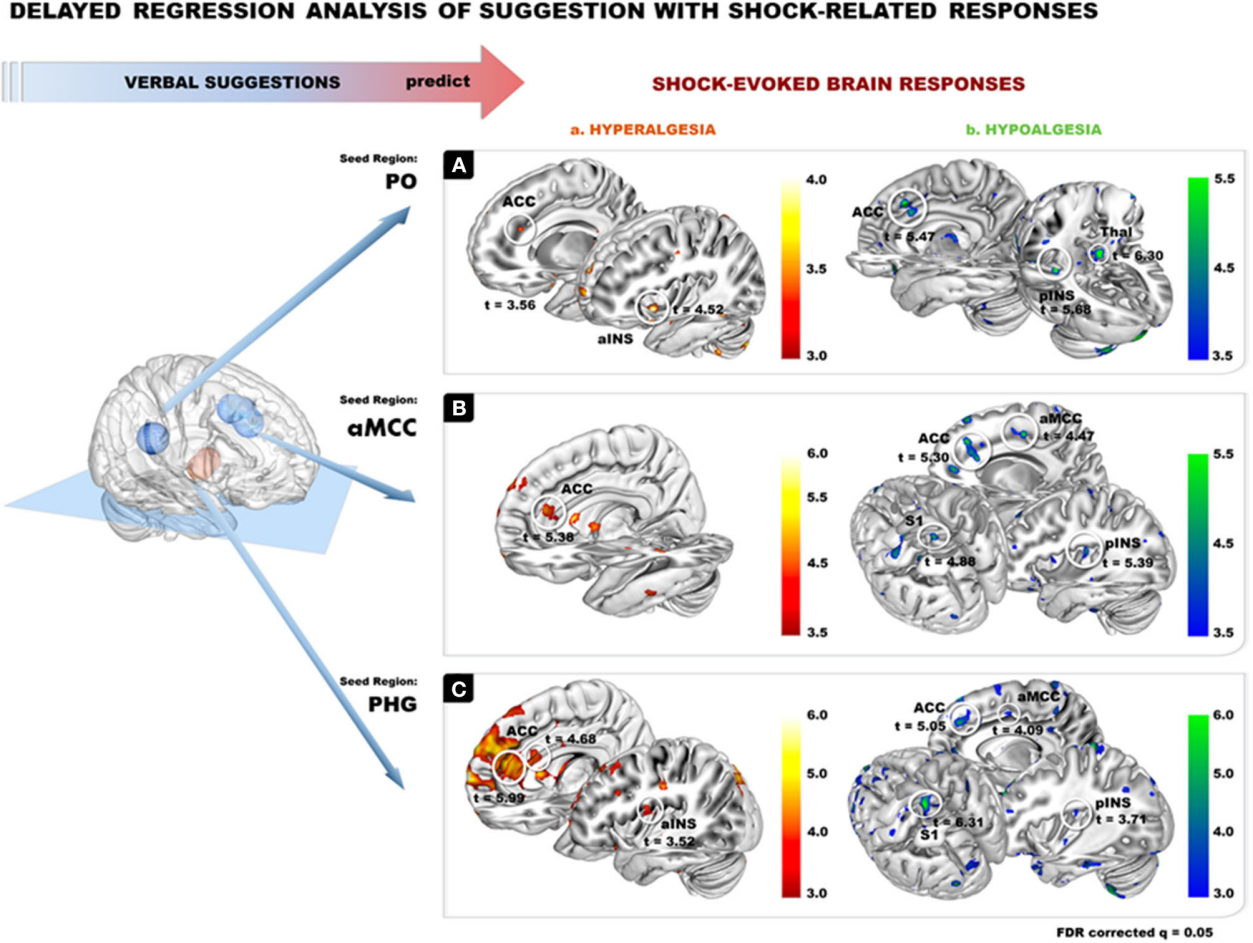
Delayed regression analysis of the 3 ROIs from suggestion of pain modulation. PO, ACC, and PHG were used as seed ROIs (10-mm radius based on the seed coordinates) to extract beta weights from the first level contrast in each subject and separately for each modulation condition (Hypo and Hyper) compared to the Neutral control, to look at the effect of suggestion in predicting brain responses to painful stimulation. **(A)** PO from suggestion of pain modulation: peaks are observed in the ACC and in the aINS during shock evoked response for Hyper condition (Aa), and for ACC, pINS and Thalamus for Hypo conditions (Ab). **(B)** AMCC from suggestion of pain modulation: peaks are observed in the ACC during shock evoked response in Hyper condition (Ba), and in rostral ACC, aMCC, pINS and S1 in Hypo condition (Bb). **(C)** PHG from suggestion of pain modulation: peaks are observed in the ACC and pINS during shock evoked response for Hyper condition (Ca.), and for ACC, aMCC, pINS and S1 for Hypo condition (Cb.). Results are reported at FDR *q* = 0.05 (see all peak and coordinates in Table 1).

In the Hyper condition, delayed regression revealed that larger deactivation in PO during suggestions predicted a larger increase in shock-evoked responses in ACC and aINS (Figure 4A(a); Table 2). In the Hypo condition, a larger deactivation of the PO during the suggestions predicted a larger decrease in shock-evoked responses in the ACC, pINS and thalamus (Figure 4A(b); Table 2).

**Table 2:** Delayed regression increase (in Hyper) and decrease (in Hypo) for ACC, PO and PHG.

**Brain area**	**Coordinates**			**Local peak *t*-value**
	***x***	***y***	***z***	
**Increase in Hyper associated with PO**				
ACC	−10	34	24	3.56
Anterior Insula (aINS)	−38	14	−6	4.52
Orbitofrontal cortex (OFC)	18	24	−20	4.79
Nucleus Accumbens (NAcc)	10	6	−10	4.89
**Decrease in Hypo associated with PO**				
ACC	6	26	38	5.47
	−4	42	24	5.05
	6	20	30	5.00
	8	10	44	4.26
	−10	14	34	3.91
Posterior Insula (pINS)	−40	−26	6	5.68
	−36	−22	16	4.56
Thalamus	12	−16	10	6.30
Putamen	26	2	6	4.54
Globus Pallidus	−10	0	0	5.71
Medial prefrontal cortex	8	50	−2	4.53
	−8	50	2	4.69
M1	−28	−24	62	4.64
**Increase in Hyper associated with aMCC**				
ACC	0	+38	16	5.38
	−10	44	12	4.45
Hippocampus (26–38 4 *t* = 4.39)	26	−38	4	4.39
**Decrease in Hypo associated with aMCC**				
ACC	−12	40	28	5.30
	−14	34	18	4.66
	12	10	44	4.46
	−14	26	28	4.21
	−4	14	36	4.17
Anterior mid cingulate cortex (aMCC)	−10	−10	46	4.47
pINS	−36	−22	16	5.39
aINS	−32	−22	16	5.39
S1	−16	−40	70	4.88
PCC	−6	−26	46	5.50
M1	−30	−24	64	5.33
SMA	−6	2	60	5.14
MPFC	−12	48	2	5.01
	−4	44	−6	4.66
Caudate	16	4	18	3.78
Putamen	28	2	8	5.37
	−26	8	−2	5.74
OFC	2	58	−14	8.00
PAG	−2	−24	−8	4.51
	0	−34	−12	4.09
**Increase in Hyper associated with PHG**				
ACC	0	20	22	4.68
	−2	38	16	5.99
	10	40	16	5.71
pINS	−38	−10	12	3.52
Medial prefrontal cortex	0	52	4	4.79
Periaqueductal gray	−20	−34	−16	4.00
Caudate	10	16	8	5.69
	−8	14	10	5.00
	8	2	12	4.78
Orbitofrontal cortex	−2	20	−26	5.14
	0	62	−18	7.28
**Decrease in Hypo associated with PHG**				
S1	−14	−36	74	6.31
ACC	−8	44	28	5.05
	6	32	22	3.45
aMCC	−8	2	44	4.09
	−2	−10	44	3.69
pINS	−34	−20	18	3.71
SMA	−2	−10	44	3.69
DLMPFC	−8	44	28	5.05
PCC	18	−56	34	5.18
	−8	−38	36	4.33
Putamen	−26	4	0	4.34
	26	12	−8	4.32
OFC	12	24	−30	6.88
	10	10	−22	5.47
	−8	50	−14	5.27
	−10	52	−14	4.55

*All effects are reported at FDR q = 0.05*.

Similarly, a larger decrease in the aMCC during suggestions predicted the modulation of shock-related responses in several shock-responsive areas in both Hyper and Hypo conditions: a larger increase in shock-evoked activation was observed in the Hyperalgesic condition in the dorsal ACC (Figure 4B(a); Table 2), while a larger decrease was found in the Hypoalgesic condition in the dorsal ACC, aMCC, pINS, and S1 (Figure 4B(b); Table 2).

Increased activity in the left PHG during the Hyperalgesic suggestions predicted a larger shock-evoked response in supracallosal ACC, rostral ACC, and pINS (Figure 4C(a); Table 2). In the Hypo condition, more activation of lPHG during the suggestions was associated with a decrease in shock-evoked responses in S1, dorsal ACC, aMCC and pINS (Figure 4C(b); Table 2).

## Discussion

The objective of this study was to explore brain regions involved in the response to verbal suggestions for hypnotic pain modulation. In line with our hypotheses, results confirmed that differential activity during suggestions predicted the modulation of brain responses to painful electric shocks.

Our findings bind verbal integration and suggestion enactment in an integrative process. This integrative process is generally explored by analyzing the end-product of modulatory effects on perceptual, cognitive and behavioral outcomes in the context of hypnotic suggestion. The protocol of this study allows the exploration of verbal suggestion encoding in the brain, in a predictive model of pain-related responses. In this section, the predictive relation of PO, aMCC and PHG with pain-evoked brain responses will be discussed first. Then, the role of the three regions implicated in the suggestion effect will be described as reflecting processes at play during verbal encoding/integration. Lastly, results will be discussed in the context of a Predictive Coding Model (PCM).

### Hypnotic Modulation of Pain-Evoked Brain Responses

The hypnosis intervention used in this study modulated pain experience effectively. Pain reports confirmed experiential changes consistent with the direction of the verbal suggestions and proportional to the individual hypnotic suggestibility score. Brain imaging results showed that suggestion-related brain activity predicted the modulation of shock-related brain responses in the thalamus, somatosensory, insular, and anterior cingulate cortex. These areas are key targets of the spino-thalamo-cortical nociceptives pathways (32, 33). The direction of the effects observed in shock-evoked responses reflected the direction of the suggestions and are thereby consistent with pain coding effects. This is in line with previous neuroimaging literature on psychological pain modulation (34–37). Modulations of the central executive, saliency, and default networks are central in the experience of hypnosis (2) and online effects of psychological pain modulation is generally anchored in attentional and anticipatory processes and can change responses to noxious stimuli in the ACC, thalamus, insula and the spinal cord, in accordance with pain report (35). Previous work further suggested that the modulation of the affective and sensory dimensions of pain could be dissociated (37, 38), with the former showing an association with changes in the aMCC (36) and the latter being associated with somatosenry activity (37). Suggestions in the present study were not designed to dissociate those two pain dimensions and results consistently show distributed modulation across several pain-activated regions (see Table 2).

Our results also suggest that despite coherent pain modulation effects, hyper- and hypoalgesic effects may engage different sub-regions of the pain-activated network. Hyperalgesic effects were found in more anterior parts of the insula [see Figures 4A(a),C(a)]. The aINS has been associated with the processing of saliency (39), prediction and error signals (40). The possibility that the hyperalgesic suggestions enhanced the saliency response is consistent with the ACC effects [see Figures 4A(a)–C(a)]. In pain context, ACC is monitoring the emotional salience of stimuli and behavioral responses, particularly in response to challenging executive function tasks (41).

Decreased shock-evoked responses in the Hypo condition is suggestive of an alteration of sensory processing. The pINS deactivation may relate more closely to a diminution of ascending sensory signals (40, 42). Similar reduction in shock-evoked responses in the aMCC, S1 and thalamus during hypoalgesia is consistent with a gating of ascending signal from the spino-thalamo-cortical pathways (32) and with the connectivity between these regions (43, 44).

Shock-related brain responses in the Hypo condition were also associated with a decrease in aMCC activity, in relation to aMCC and PHG activity during suggestions. While ACC is associated with cognitive control, aMCC is an integrative structure where affect, pain and cognitive control overlap in goal directed behavior (45).

Thalamo-cortical circuits are part of an alerting network modulated by hypnosis in relation to the experience of mental absorption (4, 13). The activity of the thalamus in shock-related response may be interpreted as an index of the top-down regulation occurring following a hypnotic induction (46). Its coactivation with pINS during Hypo conditions is also coherent with the structural connectivity of these regions in somatosensory processing (44). Taken together, these findings are coherent with the notion that hypnotic suggestions modulate brain responses to noxious stimulation.

### Suggestion-Related Brain Activity: Self-Regulation Network During Verbal Suggestions

Findings of this study also suggest that a deactivation of PO and aMCC during suggestions is predictive of the modulation of brain responses to noxious stimuli in areas that are associated with pain perception and pain modulation. The robust BOLD decrease in these regions is independent of the direction of the suggestions. This response during suggestions is interpreted in line with the role of PO and aMCC as being part of the fronto-parietal network associated with self-regulation of cognitive control and its experience (5, 47, 48).

The aMCC has a role in conflict monitoring, evaluative processes and cognitive control in goal directed behavior (45, 49). A decrease in aMCC activation may be interpreted as a decrease of need for cognitive control while passively listening to suggestions.

Participants also showed a decrease in the PO during pain modulating suggestions, where a greater decrease in PO also predicted larger suggestion effects on shock-evoked brain response. While PO is part of a network responsible for the experience of agency (47, 48), its deactivation may be interpreted as a change in this network during hypnotic suggestions. Hypnosis is generally associated with a diminution of agency experience, resulting in a feeling of automaticity, supported by the activation of PO at rest during hypnotic states (5). Nevertheless, the deactivation observed during suggestion in this study is consistent with predictions by Martin and Pacherie (50). According to them, the feeling of automaticity would not emerge during suggestions encoding, while participants have no actual information to contrast to their experience of ownership, but rather when the experience of a potential mismatch occurs and the outcome of suggestions is tested against bottom-up information (i.e., pain modulation effects, motor challenge, etc.). Research on hypnosis largely focuses on the outcomes such that previous studies did not allow to dissociate the activations related to the suggestions from those related to their actualization. This study emphasizes the critical importance to explore the temporal change of automaticity feeling along the successive stages of hypnotic processes. However, this protocol did not assess the automaticity feeling specifically following suggestions or pain stimulation, so a note of caution is in order, as we cannot assert that the activation change during suggestions was accompanied by experiential changes relevant to automaticity.

The changes observed in PO and aMCC is informative of the processes occurring while participants listen to suggestion to modulate pain and may be interpreted in accordance with their involvement in a volition and agency network (48). This network has been associated with self-regulation and perceived self-agency (5, 47), which are two fundamental aspects of hypnosis phenomenology. The deactivation in these regions would suggest a decrease in regulation and cognitive control during passive listening of verbal suggestions. However. these effects seemed to be independent of the direction of the pain modulation suggested. This was unexpected and motivated further a connectivity analysis to examine possible directional effects during the suggestions.

Functional connectivity analysis (PPI) during suggestions did not yield significant results at a conservative statistical threshold. However, more permissive exploration of the connectivity maps suggested that the amygdala was more engaged during Hyperalgesic suggestions while the NAc was more engaged during Hypoalgesic suggestions (Supplementary Table 1). The observation of such dissociation provides a basis for viewing the connectivity as a contextual priming of ulterior pain modulation. Amygdala activation is generally interpreted in relation to threat situations, fear acquisition and expression (51) and NAc activations is related to appetitively motivated behaviors, especially in ambiguous course of actions (52). Recent animal research further supports such dissociation with the ACC-NAc selectively involved in the social transfer of pain and analgesia while the ACC-Amygdala being related to the social transfer of fear (53). The results of the exploratory connectivity analysis should be considered preliminary but point to a possible differential engagement of inhibitory and facilitatory networks, consistent with the priming of directional modulatory processes during the verbal suggestions.

### Prediction of Shock-Evoked Responses

In the present study, an activity increase in lPHG during suggestions predicted the brain response to hypnotic suggestions for pain modulation. The implication of this region in contextualization is in line with classical findings in the domain of verbal memory (54) and more recent findings on verbal suggestions, where the parahippocampal complex is viewed to play a role in sustaining recent information that will modify pain experiences (18, 24).

This structure is a part of the hippocampal complex, which supports multidimensional memory codes to allow for the creation and retrieval of new associations (55). This associative process is “a conscious background state wherein subjects make unconstrained associations that are unrelated to the immediate external environment” (55). The response of the hippocampal complex in our study may reflect its role in contextual learning, memory and pain anticipation/expectations induced by verbal suggestions of pain modulation (25, 56, 57). In this interest, verbal suggestions may then be conceived as priming signals to contextualize aversive inputs in favor of hyperalgesia or hypoalgesia. In order to investigate the top-down effect of verbal suggestions on perception, Cayol and Nazir (10) proposed thinking of language comprehension as the output of an emulator. This emulator serves to model the situation described verbally by providing stored contextual information. Consistent with its function in contextual association (25), the PHG can be described as part of this emulator. The activation of PHG during suggestions as a predictor of pain-related brain activity in Hyper and Hypo appear as a marker of this ability to contextualize and influence pain perception.

The idea that the brain contextualizes inputs with previous semantic information, allowing us to verify, predict and prepare our perceptions of the environment, could be viewed in light of the Predictive Coding Model (PCM). The PCM can be used as a framework to help elucidate how words processing can exert pain modulation. Frith and Friston (58) described a system in which expectations are integrated at different levels of the neuro-axis according to the a priori of the dominant levels. This model can be applied to many psychological effects and has recently been applied to embodied language theories (10) and pain perception and modulation (59). Based on this model, verbal suggestion biases perception in a top-down fashion.

Hypnosis is based on verbal suggestions to enter into a new state (induction) and to feel and behave according to implicit or explicit suggestions. According to a predictive coding framework, “hypnotisability is a function of the gain set on priors” (50). The impact of the hypnosis induction may be to reduce the relative precision of sensory inputs (weights assigned to bottom-up signals) and prediction errors, while focusing attention on verbally suggested priors. This context may, in turn, facilitate the integration of verbal suggestions as informative of this emulator.

The notion of emulation refers not only to a sensorimotor preparation, but also to the ability of the brain to run an off-line model in order to produce mental imagery (60), a conscious access to simulations of emulators, being engaged in hypnosis (2). The focus of attention on this experience would act by weighing in favor of the suggested prior when a subsequent perception would call for contextualization. Verbal suggestions a priori would act at a higher level of integration than bottom-up nociception. The impact of the fronto-parietal network deactivation would be to reduce cognitive surveillance of bottom-up signals and prediction errors, while allowing focus on verbally suggested prior.

Not everyone weights words in the same way. In the PCM framework, both top-down and bottom-up processes inform this emulator. People who are able to add weight to the top-down predictions in the emulation of perception, using verbal suggestions, will benefit from being able to change their experience. As such, the contextual association of which PHG is an essential contributor would be more effectively used in a top-down fashion in individuals benefitting from pain modulation suggestions. The lPHG activity during suggestions may represent the degree of integration of the verbal suggestions- or the weight it is gaining, in a PCM- in preparation for informed ulterior perception. The ability to anchor pain perception in the context induced by verbal suggestions would be equivalent to a prioritization of the top-down association, resulting in more pain modulation according to the direction of pain modulatory suggestions.

### Study Limitations

This study has many strengths and its share of limitations. The protocol allowed the study of brain correlates of suggestion encoding as predictors of shock-evoked responses. Results were obtained in three conditions (Hypo, Hyper and Neutral). This choice is informative of pain modulation in general, rather than being specific to hypnotic hypoalgesia, the focus of most previous studies. The study design did not allow us to explore mediation models predicting changes in pain perception from stimulus-evoked brain responses because we only obtained pain ratings after blocks of six stimuli in the control conditions and blocks of nine stimuli in the pain modulation conditions (see Figure 1). Future research designs should attempt to integrate pain reports on a trial-by-trial basis to allow testing more complex models.

Importantly, the inclusion of a Hyper condition allowed the demonstration of large non-directional effects during the verbal suggestions and directional (i.e., pain-coding) effects of suggestions in shock-evoked responses. This distinction of the neural activity related to verbal suggestion encoding and to pain modulation during shocks provides a fundamental milestone in the description of hypnosis phenomenology.

However, we cannot fully assert the role of hypnosis in this model integrating verbal suggestion in a predictive coding framework. Without a baseline BOLD scan, it cannot be inferred that the observed activation changes are explained by the hypnotic induction. Suggestions effects may also be observed in a variety of context not involving hypnosis and the present results may therefore be important for, but not specific to, hypnosis.

Furthermore, one cannot assume that the effects observed here would generalize to a non-pain context. It is possible that regions used as predictor (aMCC, PO, and PHG) would not be found in a condition that is not pain-related. Nevertheless, these three regions have been associated with self-monitoring and self-regulation [aMCC; (45, 49), hypnotic automaticity and perceived self-agency [PO and aMCC; (5, 47, 48)], and contextual learning and expectations [PHG; (25, 55–57)]. In view of previous research, the generalization to other contexts appears to be plausible but should be tested more explicitly in studies contrasting pain-related suggestions to other types of suggestions. In the same interest, this study examined hypnotic suggestions to modulate pain, but suggestions effects may be observed in a variety of contexts not explored here. The present results may therefore be important for, but not specific to, hypnosis and pain modulation.

## Conclusion

This study provides a basis to further explore the transformation of verbal suggestions into perceptual modulatory processes. Results are congruent with a pain modulation effect following verbal suggestion, and inform models of language and hypnosis in a PCM framework of the experience of pain modulation.

A pain modulation effect was observed in Hyper and Hypo conditions in line with the direction of the suggestions. The functional relationship between verbal suggestion processing and modulation in the second stage is consistent with the fact that brain processing of verbal suggestions in the context of hypnosis predicts the ability to modulate pain-evoked responses. Greater deactivation of regions related to self-regulation and agency (aMCC and PO) during passive listening of suggestions was related to greater pain modulation, subsequently. The processes involved in the suggestions occurred in brain regions related to pain, but differed depending on the direction of the modulation. The increase and decrease of pain appear to be based on different mechanisms. The ability to modulate pain during shock response is also related to activations of the lPHG during suggestions, a region related to contextualization and verbal memory. Individuals showing greater activation of lPHG during suggestions encoding, displayed more modulation in brain response to the painful shocks. The individual ability to modulate pain following hypnotic suggestions may be considered to reflect the activity of an internal emulator integrating verbal information to generate predictions which will override bottom-up signals to alter the experience of pain.

This study proposes a possible neurobiological framework for the integration of verbal suggestions into an internal model which facilitates experiential changes as one interacts with the outside world. Such approach may be applicable to a variety of language-based biopsychosocial interventions.

## Data Availability Statement

The datasets presented in this article are not readily available because we do not have the permission to share individual data sets but activation maps can be shared upon request. Requests to access the datasets should be directed to Pierre Rainville, pierre.rainville@umontreal.ca.

## Ethics Statement

The studies involving human participants were reviewed and approved by Comité d'éthique de la recherche — Vieillissement et neuroimagerie. The patients/participants provided their written informed consent to participate in this study.

## Author Contributions

AS, BH, MP, and PR conceived the study. AS planned the experiment, performed the hypnotic induction, and assessed the suggestibility of each participant individually using the SHSS-A. AS acquired data with BH and J-IC. CD, J-IC, and PR analyzed the brain imaging data. CD updated the literature review and contributed to the interpretation of the results, with J-IC and PR. CD, J-IC, and PR wrote the paper. All authors provided feedback on or approved the final version of the manuscript.

## Funding

This work was funded by the grants from Canadian Institutes of Health Research (grants no: MOP 130341).

## Conflict of Interest

The authors declare that the research was conducted in the absence of any commercial or financial relationships that could be construed as a potential conflict of interest.

## Publisher's Note

All claims expressed in this article are solely those of the authors and do not necessarily represent those of their affiliated organizations, or those of the publisher, the editors and the reviewers. Any product that may be evaluated in this article, or claim that may be made by its manufacturer, is not guaranteed or endorsed by the publisher.

## Abbreviations

PCM: Predictive Coding Model
aMCC: anterior medial cingulate cortex
PO: Parietal operculum
aINS: anterior insula
pINS: posterior insulta
aMCC: anterior mid-cingulate cortex
Hypo: hypoalgesia
Hyper: hyperalgesia.
