# Supplementary material for: Brain Responses to Hypnotic Verbal Suggestions Predict Pain Modulation
Source: Front Pain Res (Lausanne). 2021 Dec 23;2:757384. doi: 10.3389/fpain.2021.757384 (PMC8915547; doi:10.3389/fpain.2021.757384)
Supplement: Supplementary file 1 [file Table_1.DOCX]

**Supplementary Table 1**. Functional connectivity during suggestions (PPI analysis)

| **BRAIN AREA** | **COORDINATES** | | | **LOCAL PEAK t-value** |
| --- | --- | --- | --- | --- |
|  | ***x*** | ***y*** | ***z*** |  |
|  |  |  |  |  |
| **Association with lPHG in Hypo** |  |  |  |  |
| Nucleus accumbens * | -8 | 6 | 0 | 5.28 |
|  | 4 | 6 | 0 | 4.40 |
| Inferior frontal | -62 | 8 | 6 | 5.14 |
| Superior Parietal | 26 | -58 | 60 | 4.58 |
| Dorsolateral prefrontal cortex | 46 | 16 | 16 | 4.23 |
| Anterior cingular cortex | -4 | 22 | 18 | 4.43 |
| Occipital lobe | -12 | -76 | 18 | 5.06 |
|  |  |  |  |  |
| **Association with lPHG in Hyper** |  |  |  |  |
| Amygdala* | 20  24 | -2  0 | -16  -18 | 4.25  4.09 |
| Posterior parietal | -60 | -56 | 44 | 6.51 |
| Superior temporal lobe | -58 | -2 | 2 | 4.22 |
| Superior temporal gyrus | 32 | 10 | -20 | 4.47 |
| Temporal pole | 30  -48 | 16  -8 | -40  -38 | 4.75  4.56 |
| Thalamus | 12 | -32 | 4 | 4.03 |
|  |  |  |  |  |

p = 0.001 uncorrected with k>5

* = reported in results section
