# Supplementary material for: Brain Responses to Hypnotic Verbal Suggestions Predict Pain Modulation
Source: Front Pain Res (Lausanne). 2021 Dec 23;2:757384. doi: 10.3389/fpain.2021.757384 (PMC8915547; doi:10.3389/fpain.2021.757384)
Supplement: Supplementary file 2 [file Data_Sheet_1.docx]

**Supplementary material**

Verbatim- Hypnotic suggestions were delivered in french

NEUTRAL 01 (94 sec) - fr

Tes sensations sont tout à fait normales.

Restes bien détendu.

Détends-toi.

Détends-toi complètement

Détends chacun des muscles de ton corps.

Détends les muscles de tes jambes. Détends les muscles de tes pieds… Détends les muscles de tes bras, de tes doigts… Détends les muscles de ton cou, de ta poitrine, détends tous les muscles de ton corps.

Ta cheville et ton pied sont tout à fait normaux et ont leur constitution normale d'os, de muscles et de chair. Ta peau a sa sensibilité habituelle

Maintenant, laisses ton corps devenir mou, mou, mou

Détends-toi de plus en plus.

Détends-toi complètement.

Tes sensations sont tout à fait normales.

Les stimulations vont reprendre maintenant.

NEUTRAL 01 (94 sec) - en

Your sensations are completely normal.

Stay relaxed.

Relax.

Relax completely.

Relax every muscle in your body.

Relax the muscles in your legs. Relax the muscles in your feet... Relax the muscles in your arms, in your fingers... Relax the muscles in your neck, in your chest, relax every muscle in your body.

Your ankle and your foot are completely normal and have their normal constitution of bone, muscle and flesh. Your skin has its usual sensitivity

Now let your body become soft, soft, soft.

Relax, more and more.

Relax completely.

Your sensations are completely normal.

Stimulation will resume now.

NEUTRAL 2 (107 sec)

Tes sensations vont maintenant redevenir tout à fait normales.

Restes bien détendu.

Détends-toi.

Détends-toi complètement.

Détends chacun des muscles de ton corps.

Détends les muscles de tes jambes.

Détends les muscles de tes pieds.

Détends les muscles de tes bras, de tes doigts.

Détends les muscles de ton cou, de ta poitrine.

Détends tous les muscles de ton corps.

Ta cheville n'est plus engourdie et ton pied retrouve sa constitution normale d'os, de muscles et de chair.

Ta peau a retrouvé sa sensibilité habituelle.

Maintenant, laisses ton corps devenir mou, mou, mou.

Détends-toi de plus en plus.

Détends-toi complètement.

Tes sensations redeviennent tout à fait normales.

Les stimulations vont reprendre maintenant.

NEUTRAL 2 (107 sec)- en

Your sensations will now return to normal.

Stay relaxed.

Relax.

Relax completely.

Relax every muscle in your body.

Relax the muscles in your legs.

Relax the muscles in your feet.

Relax the muscles in your arms, in your fingers.

Relax the muscles in your neck, your chest.

Relax all the muscles in your body.

Your ankle is no longer numb and your foot is back to its normal constitution of bone, muscle and flesh.

Your skin has regained its normal sensitivity.

Now let your body become soft, soft, soft.

Relax, more and more.

Relax completely.

Your sensations are returning to normal.

Now the stimulation will start again.

HYPOALGESIA 1 (128 sec) - fr

Maintenant que tu es bien installé : Détends-toi.

Détends-toi complètement, profondément…

Détends chacun de tes muscles, de ton corps.

Tu vas maintenant vivre une expérience surprenante

Laisses ton corps devenir mou, mou, mou.

Imagines maintenant que ta cheville est en train de devenir de moins en moins sensible.

Ta peau devient comme engourdie et tu sentiras à peine les stimulations.

Un peu comme une couche de caoutchouc s'interposait entre ta peau et les stimulations.

Cette couche devient de plus en plus épaisse, comme si ta peau elle-même se transformait imagines-toi que ton pied complet se transforme en caoutchouc.

Comme tu le sais peut-être, le caoutchouc est un bon isolant.

Quand les stimulations reprendront, tu seras peut-être étonné de constater que les chocs te semblent beaucoup moins intense…

Qu'ils pincent moins, qu'ils chauffent moins, qu'ils picottent moins ou encore que tu ne les ressens plus du tout.

Prends le temps de bien t'imaginer ton pied complètement en caoutchouc.

Et tu constateras que ta cheville sera de moins en moins sensible.

Les stimulations vont reprendre, maintenant.

HYPOALGESIA 1 (128 sec)- en

Now that you are comfortable: Relax.

Relax completely, deeply...

Relax every muscle in your body.

You are now going to have a surprising experience.

Let your body become soft, soft, soft.

Imagine now that your ankle is becoming less and less sensitive.

Your skin becomes numb and you will hardly feel any stimulation.

It's like a layer of rubber between your skin and the stimuli.

This layer becomes thicker and thicker, as if your skin itself were turning into rubber.

As you may know, rubber is a good insulator.

When the stimuli start again, you may be surprised to find that the shocks seem much less intense...

That they don't pinch as much, that they don't get as hot, that they don't tingle as much or that you don't feel them at all.

Take the time to imagine your foot completely made of rubber.

You will notice that your ankle will be less and less sensitive.

Stimulation will resume now.

HYPOALGESIA 2 (119 sec)- fr

Je te demande encore une fois de rester bien détendu.

Détends-toi complètement, profondément.

Détends chacun de tes muscles de ton corps.

Tu vas maintenant vivre une expérience surprenante.

Laisse ton corps devenir mou, mou, mou.

Imagines maintenant que ta cheville est de nouveau en train de devenir de moins en moins sensible.

Ta peau devient comme engourdie.

Et tu sentiras à peine les stimulations. Un peu comme si une couche de caoutchouc s'interposait entre ta peau et les stimulations.

Cette couche devient de plus en plus épaisse, comme si ta peau elle-même se transformait.

Imagines-toi que ton pied complet se transforme en caoutchouc. Rappelle-toi que le caoutchouc est un bon isolant.

Quand les stimulations reprendront, tu seras peut-être étonné de constater que les chocs te semblent beaucoup moins intense. Qu'ils pincent moins, qu'ils chauffent moins, qu'ils picotent moins, ou encore que tu ne les ressens plus du tout.

Prends le temps de bien t'imaginer ton pied complètement en caoutchouc et tu constateras que ta cheville sera de moins en moins sensible.

Les stimulations vont reprendre, maintenant.

HYPOALGESIA 2 (119 sec)- en

I ask you once again to stay very relaxed.

Relax completely, deeply.

Relax every muscle in your body.

You are now going to have a surprising experience.

Let your body become soft, soft, soft.

Imagine now that your ankle is again becoming less and less sensitive.

Your skin becomes numb.

And you will hardly feel any stimulation. It's as if a layer of rubber was placed between your skin and the stimuli.

This layer becomes thicker and thicker, as if your skin itself is changing.

Imagine that your whole foot is turning into rubber. Remember that rubber is a good insulator.

When the stimuli start again, you may be surprised to find that the shocks seem much less intense. They don't pinch as much, they don't get as hot, they don't tingle as much, or you don't feel them at all.

Take the time to imagine your foot completely made of rubber and you will notice that your ankle will be less and less sensitive.

Stimulation will resume now.

HYPERALGESIA (122 sec)- fr

Maintenant que tu es bien installé, détends-toi.

Détends-toi complètement, profondément.

Détends chacun de tes muscles, de ton corps.

Tu vas maintenant vivre une expérience surprenante.

Laisse ton corps devenir mou, mou, mou.

Imagine maintenant que ta cheville est en train de devenir de plus en plus sensible.

Ta peau devient très sensible et tu sentiras de plus en plus les stimulations.

Un peu comme si une couche de métal s'interposait entre ta peau et les stimulations.

Cette couche devient de plus en plus épaisse…

Comme si ta peau elle-même se transformait.

Imagines-toi maintenant que ton pied complet se transforme en métal.

Comme tu le sais peut-être, le métal est un très bon conducteur.

Quand les stimulations reprendront, tu seras peut-être étonné de constater que les chocs te semblent amplifiés.

Qu'ils semblent beaucoup plus intense, qu'ils pincent plus, qu'ils chauffent plus ou encore qu'ils picottent plus.

Prends le temps de bien t'imaginer ton pied complètement en métal et tu constateras que ta cheville sera de plus en plus sensible.

Les stimulations vont reprendre, maintenant.

HYPERALGESIA (122 sec)- en

Now that you are comfortable, relax.

Relax completely, deeply.

Relax every muscle in your body.

You are now going to have a surprising experience.

Let your body become soft, soft, soft.

Now imagine that your ankle is becoming more and more sensitive.

Your skin is becoming very sensitive and you will feel more and more stimulation.

It's as if a layer of metal was placed between your skin and the stimuli.

This layer becomes thicker and thicker...

As if your skin itself was changing.

Now imagine that your whole foot is turning into metal.

As you may know, metal is a very good conductor.

When the stimulation starts again, you may be surprised to find that the shocks seem to be amplified.

That they seem much more intense, that they pinch more, that they heat up more or that they tingle more.

Take the time to imagine your foot completely made of metal and you will notice that your ankle will be more and more sensitive.

The stimulation will start again now.

HYPERALGESIA 2 (117 sec)- fr

Je te demande encore une fois de rester bien détendu.

Détends-toi. Détends-toi complètement, profondément.

Détends chacun de tes muscles de ton corps.

Tu vas maintenant vivre une expérience surprenante.

Laisses ton corps devenir mou, mou, mou.

Imagines maintenant que ta cheville est de nouveau en train de devenir de plus en plus sensible.

Ta peau devient très sensible et tu sentiras de plus en plus les stimulations un peu comme si une couche de métal s'interposait entre ta peau et les stimulations.

Cette couche devient de plus en plus épaisse, comme si ta peau elle-même se transformait.

Imagines-toi maintenant que ton pied complet se transforme en métal

Rappelle-toi que le métal est un très bon conducteur.

Quand les stimulations reprendront tu seras peut-être étonné de constater que les chocs te semblent amplifiés.

Qu'ils semblent beaucoup plus intenses.

Qu'ils pincent plus, qu'ils chauffent plus ou encore qu'ils picotent plus.

Prends-le temps de bien t'imaginer ton pied complètement en métal et tu constateras que ta cheville sera de plus en plus sensible.

Les stimulations vont reprendre, maintenant.

HYPERALGESIA 2 (117 sec)- en

I ask you once again to stay relaxed.

Just relax. Relax completely, deeply.

Relax every muscle in your body.

Now you are going to have an amazing experience.

Let your body become soft, soft, soft.

Now imagine that your ankle is again becoming more and more sensitive.

Your skin is becoming very sensitive and you will feel the stimuli more and more, as if a layer of metal was placed between your skin and the stimuli.

This layer becomes thicker and thicker, as if your skin itself were changing.

Now imagine that your whole foot is turning into metal

Remember that metal is a very good conductor.

When the stimulation starts again, you may be surprised to find that the shocks seem to be amplified.

That they seem much more intense.

That they pinch more, that they heat up more or that they tingle more.

Take the time to imagine your foot completely made of metal and you will notice that your ankle will be more and more sensitive.

The stimulation will start again now.
