# Supplementary figures and images for: Brain Responses to Hypnotic Verbal Suggestions Predict Pain Modulation
Source: Front Pain Res (Lausanne). 2021 Dec 23;2:757384. doi: 10.3389/fpain.2021.757384 (PMC8915547; doi:10.3389/fpain.2021.757384)

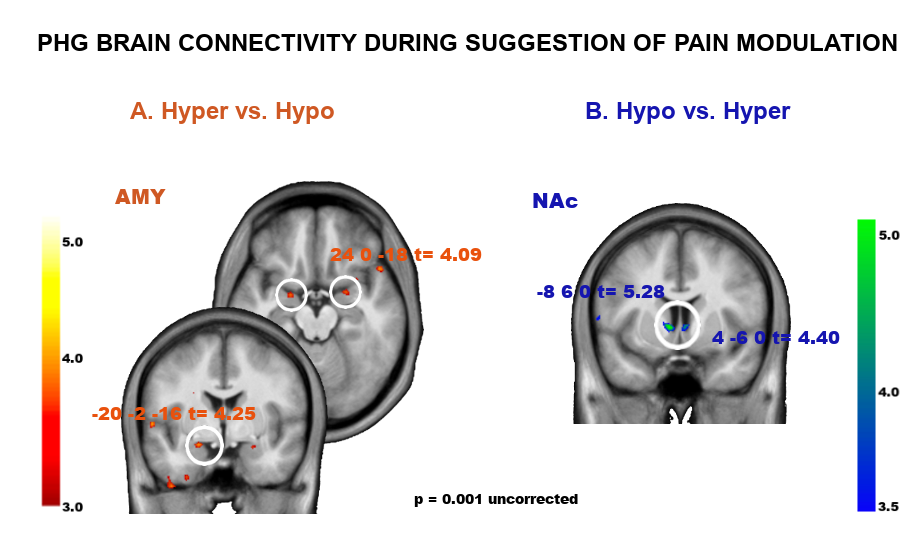

Supplement: Supplementary file 3 [file Image_1.TIF]
